# Defect structure evolution of polyacrylonitrile and single wall carbon nanotube nanocomposites: a molecular dynamics simulation approach

**DOI:** 10.1038/s41598-020-68812-7

**Published:** 2020-07-16

**Authors:** So Jeong Heo, Kwang Ho Kim, Byungchan Han, Han Gi Chae, Seung Geol Lee

**Affiliations:** 1 School of Materials Science and Engineering, Ulsan National Institute of Science and Technology, 50 Unist-gil, Ulsan, 44919 Republic of Korea; 2 Department of Organic Material Science and Engineering, Pusan National University, 2, Busandaehak-ro 63beon-gil, Geumjeong-gu, Busan, 46241 Republic of Korea; 3 School of Materials Science and Engineering, Pusan National University, 2, Busandaehak-ro 63 Beon-gil, Geumjeong-gu, Busan, 46241 Republic of Korea; 4 Department of Chemical and Biomolecular Engineering, Yonsei University, 50 Yonsei-ro, Seodaemun-gu, Seoul, 03722 Republic of Korea

**Keywords:** Engineering, Materials science

## Abstract

In this study, molecular dynamics simulations were performed to understand the defect structure development of polyacrylonitrile-single wall carbon nanotube (PAN-SWNT) nanocomposites. Three different models (control PAN, PAN-SWNT(5,5), and PAN-SWNT(10,10)) with a SWNT concentration of 5 wt% for the nanocomposites were tested to study under large extensional deformation to the strain of 100% to study the corresponding mechanical properties. Upon deformation, the higher stress was observed in both nanocomposite systems as compared to the control PAN, indicating effective reinforcement. The higher Young’s (4.76 ± 0.24 GPa) and bulk (4.19 ± 0.25 GPa) moduli were observed when the smaller-diameter SWNT_(5,5)_ was used, suggesting that SWNT_(5,5)_ resists stress better. The void structure formation was clearly observed in PAN-SWNT_(10,10)_, while the nanocomposite with smaller diameter SWNT_(5,5)_ did not show the development of such a defect structure. In addition, the voids at the end of SWNT_(10,10)_ became larger in the drawing direction with increasing deformation.

## Introduction

Carbon nanotube (CNT)-based polymer nanocomposites have been extensively studied for more than 20 years^1^. A number of researches on CNT-based polymer nanocomposites have shown the promising potential of CNTs as a reinforcing material and as a source of multiple functionalities owing to the strong interaction with polymer matrices^2–9^. Bhattacharyya et al.^2^ investigated the enhanced interfacial adhesion between the polyamide12-matrix and styrene–maleic-anhydride copolymer encapsulated (SMA-encapsulated) single-wall carbon nanotubes (SWNTs), showing that tensile and dynamic mechanical properties of the composites were improved. In addition to the bulk composites, CNT-based polymer nanocomposites have often been processed into fibers because the fibrous shape is the best way to fully exploit the anisotropic structure of CNTs and polymers. Among various types of polymer-CNT nanocomposite fiber, polyacrylonitrile (PAN) has been among the most studied polymeric system^10–12^. Li et al.^3^ studied the effect of drawing on the mechanical properties of the PAN/multi-wall carbon nanotube (MWNT) composite fiber. Chae et al. investigated enhancing PAN-CNT composites by gel-spinning^4,5^ and CNT-exfoliation^6^ processes, and Wang et al.^7^ showed that the mechanical properties of PAN-CNT nanocomposites were enhanced by the orientation of nanocomposite CNTs during deformation. Since the atomistic level of simulations such as molecular dynamics (MD) can provide detailed information of the nanocomposites in molecular level, a few studies regarding on PAN-CNT nanocomposites was also conducted using MD simulations^13,14^. Meng et al.^13^ investigated the effect of nanotube dispersion and polymer conformational confinement on interaction PAN-SWNT interaction energies using full atomistic molecular dynamics (MD) computational simulations with experiments. Gissinger et al.^14^ have investigated structure–property relationships in PAN-SWNT composites as a function of polymer crystallinity and different types of CNTs through molecular dynamics (MD).

Unlike bulk composites, fiber spinning includes a drawing process that improves molecular alignment and, as a result, improves mechanical properties of fibers^15–18^. However, it is important to note that molecular slippage between polymer chains and CNTs may create defect structures at both ends of the CNTs in polymer-CNT nanocomposite fibers, resulting in the reduced mechanical properties of nanocomposite fibers. Jain et al.^19^ observed the elongated void structure formation in the PAN-MWNT fiber at the end of MWNT, which was named as ‘edge effect’. In addition, studies of the relationship between void structure and mechanical properties of PAN-CNT nanocomposites have also been reported^20^. Although void structure formation may adversely affect on the resulting properties of nanocomposites, no significant deterioration of mechanical properties has been observed, which may be due to the ductile nature of polymer matrix and/or because the strong interaction between polymer matrix and CNT may overcome the effect of the void. However, it has to be considered that such a void structure may play a critical role in the case of the brittle matrix. PAN polymer is a well-known precursor for carbon fiber, and recent studies have shown that even nanometer-scale defects can be of critical failure site^21^. A number of studies^2–7^ on PAN/CNT composite fibers have been conducted for the last two decades to improve carbon fiber properties further. Therefore, it is essential to understand the role of CNT for the defect structure development in the precursor fiber processing stage. Since understanding the mechanism of molecular alignment and defect-structure formation in PAN-CNT nanocomposite fibers upon drawing is important, we expect that the atomistic simulations can provide the detailed mechanisms on the effect of void formation in PAN-CNT nanocomposite. It is also important to understand how strong the interaction between PAN molecules and CNTs is and the effect of CNT type. Nonetheless, it is difficult to observe these behaviors experimentally and understand physical interaction of CNT with PAN.

In this study, a computer simulation study based on molecular dynamics (MD) was conducted to clearly show the effect of CNT on the void structure formation under large deformation, which mimics the drawing process in fiber manufacturing. The analysis was used to investigate the effect of CNT and void structure on the mechanical properties of nanocomposites. As noted earlier, the different types of CNTs were also incorporated for calculation, and the difference in microstructure evolution was investigated.

## Computational details

### Force field and molecular dynamics

**Figure 1:**
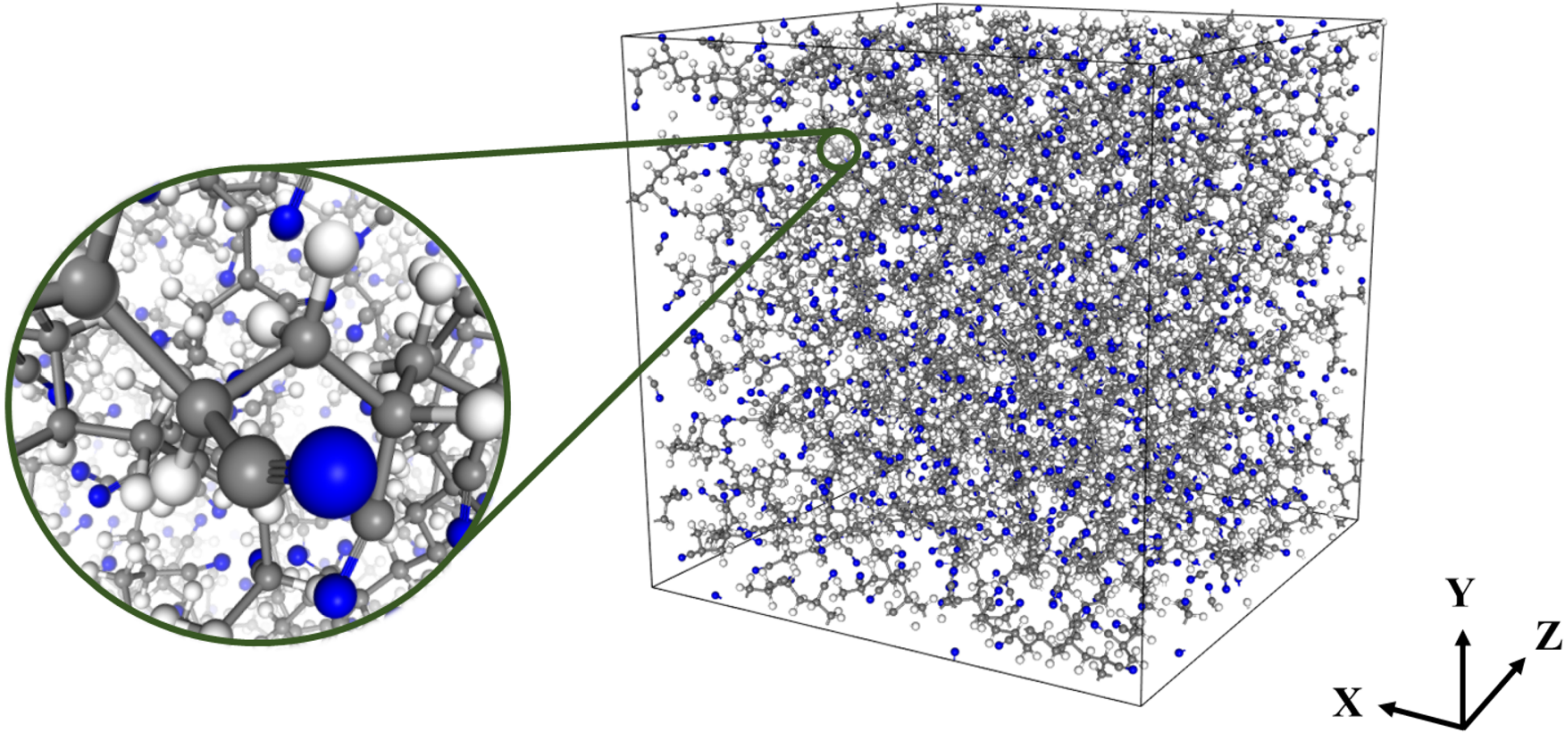
Model of the PAN-matrix under periodic boundary conditions. White, gray and blue colored circles represent hydrogen, carbon and nitrogen atoms, respectively.

Molecular dynamics (MD) calculations were used to investigate structural evolution during extension, as well as the mechanical properties of the polymer-CNT nanocomposites. A DREIDING force field was used to describe the bonded and non-bonded interactions of the PAN-CNT nanocomposite models for all calculations. We used modified van der Waals parameters as shown in Table 1. The DREIDING force field^22^ is known to be reasonable for predicting the structures and dynamics of organic and main-group inorganic molecules^23–28^. The total energy ($E_{total}$) was calculated according to Eq. (1):

1 $$E_{total} = E_{vdw} + E_{Q} + E_{bond} + E_{angle} + E_{torsion} + E_{inversion} ,$$

where $E_{vdw}$, $E_{Q}$, $E_{bond}$, $E_{angle}$, $E_{torsion}$, and $E_{inversion}$ are the van der Waals, electrostatic, bond-stretching, angle-bending, torsion, and inversion energy components, respectively. The atomic charges of PAN and SWNT were assigned from Mulliken population analyses^29^ at the DNP level using the generalized gradient approximation (GGA) of the Perdew-Burke-Ernzerhof (PBE) functional^30^. The schemes of partial charges for PAN and SWNT were shown in Fig. 1S. All MD simulations were conducted using the Large-scale Atomic/Molecular Massively Parallel Simulator (LAMMPS)^31^. The equations of motion were integrated using a velocity Verlet algorithm^32^ with a time step of 1.0 fs; a damping relaxation time of 0.1 ps was also employed.

**Table 1:** van der Waals parameters based on Lennard–Jones potential form. (*E*_*vdW*_ = *D*_0_[(*R*_0_/*R*)^12^ − 2(*R*_0_/*R*)^6^]).

	R_0_ (Å)	D_0_ (kcal/mol)
H (PAN)	2.9070	0.0202
C (PAN)	3.5469	0.1263
N (PAN)	3.3320	0.1028
H (CNT)	2.9070	0.0202
C (CNT)	3.8050	0.0692

### Model construction

#### PAN-matrix model

PAN polymer chain composed of 100 acrylonitrile (AN) units were used to construct the PAN matrix. A total of 10 PAN polymer chains were used in this investigation. Periodic boundary conditions (PBCs) were used. The atomic visualizer used in this study was Materials Visualizer in Materials Studio software^33^. As shown in Fig. 1, the initial unit cell was cubic, with x, y, and z-axis lengths of 41.881 Å, and was constructed using Monte Carlo (MC) simulation by Amorphous module in Materials Studio software^33^. Monte Carlo methods are a broad class of computational algorithms that rely on repeated random sampling to obtain energetically stable configurations of model systems. First, volume annealing^34,35^ was applied to loosen the stress-like constraints in the PAN polymer chains efficiently. Total three cycles of volume annealing through sequential thermal (between 300 and 600 K) and pressure (between densities of 0.6 and 1.2) were conducted to the model systems. Consequently, three cycles of temperature annealing were also performed up to 1,000 K while maintaining a constant cell volume to escape local minima quickly. Lastly, the isothermal-isobaric (NPT) ensemble was then used to equilibrate the PAN matrix for 5 ns at 298 K. The final density of the PAN matrix obtained in this manner was 1.177 ± 0.004 g/cm^3^, which is well-matched with the experimental value for isotropic PAN (1.18 g/cm^3^)^36,37^.

#### SWNT model

Infinite or capped SWNT models have generally been used to reinforce polymer matrices^38,39^. The properties of an SWNT depend on its structure, such as its chirality and the number of SWNT walls present^40–42^. Among various types of SWNT, we chose a single-wall nanotube (SWNT) that exhibits arm-chair chirality, because this type of SWNT has superior elongation properties^42^ and a good reinforcement efficiency^43^. Hence, in order to investigate the effect of the SWNT diameter on the overall structural-evolution mechanism and mechanical properties of the fiber, we constructed single-walled (5,5) and (10,10) armchair SWNTs (SWNT_(5,5)_ and SWNT_(10,10)_) with diameters of 7.404 Å and 14.375, respectively. Both SWNT models were 27.837 Å in length and were terminated with hydrogens and open at each end. The length of SWNT models was set up without consideration of the length effect on the void formation in this study. Also, due to the limitation of the model size on MD simulations, we choose the length of the SWNT model. In this study, we have added only one CNT for each system to solely investigate the defect structure formation in the vicinity of CNTs and the corresponding physical properties of nanocomposites for the simplicity of calculation.

#### PAN-SWNT nanocomposite model

**Figure 2:**
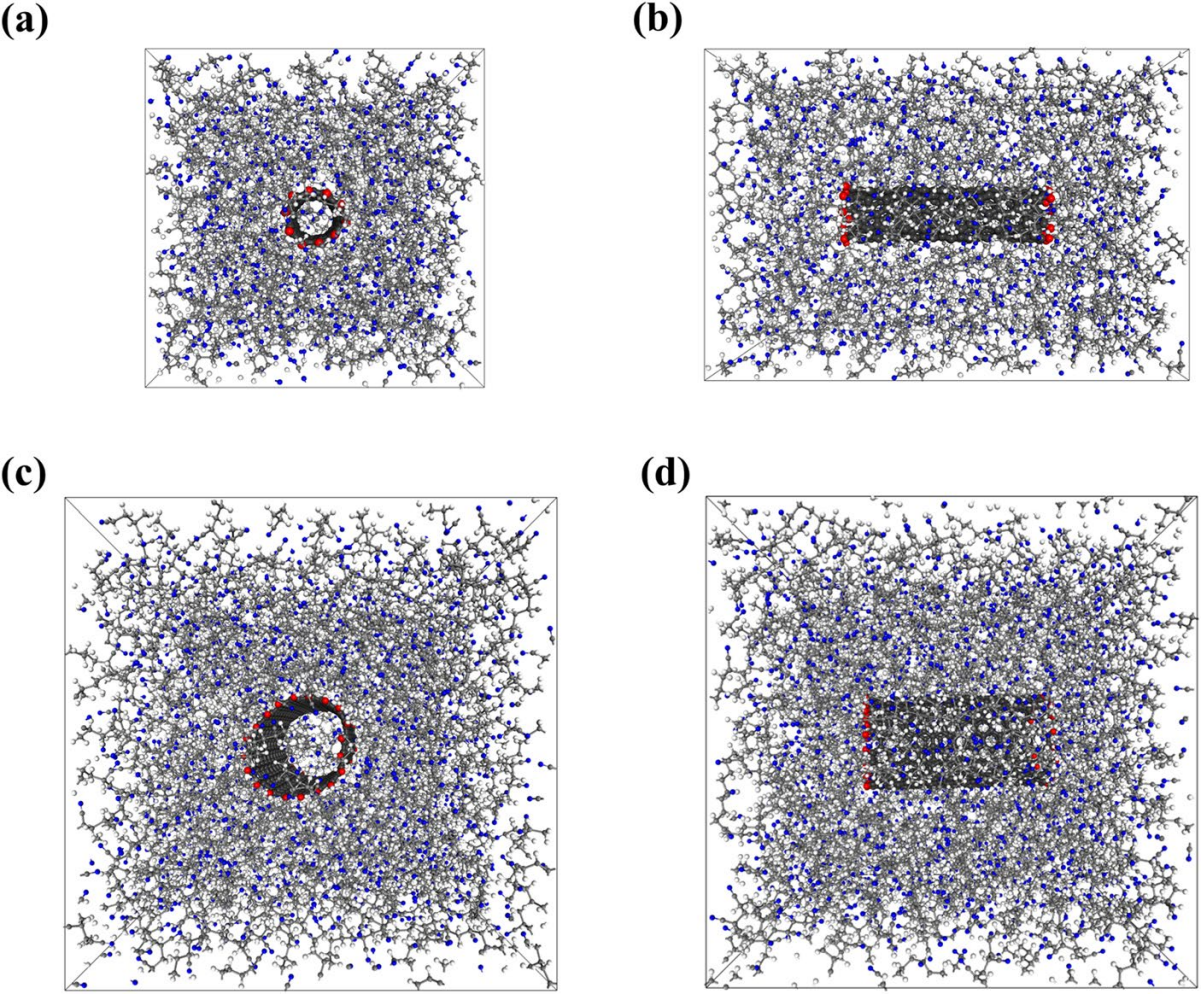
(**a**, **b**) Front and side views of the PAN-SWNT_(5,5)_ nanocomposite. (**c**, **d**) Front and side views of PAN-SWNT_(10,10)_ nanocomposite. White, gray, and blue colored circles represent the hydrogen, carbon, and nitrogen atoms of PAN, respectively; red and black colored circles represent the hydrogen and carbon atoms of the SWNT, respectively.

The weight fraction of SWNT_(5,5)_ or SWNT_(10,10)_ in the PAN matrix was set to 5 wt% on the basis of an experimental report^7^. The PAN-SWNT_(5,5)_ system was constructed with a tetragonal unit cell with x- and y-axis lengths of 37.794 Å, and a z-axis length of 54 Å, as shown in Fig. 2a, b. In order to ensure the same weight fraction of the PAN-SWNT_(10,10)_ system, PBC dimensions of 53.448 Å (x- and y-axes) and 54 Å (z-axis) were employed, as shown in Fig. 2c, d. Volume and temperature annealing were then performed in order to equilibrate the nanocomposite system. Following annealing, an NPT run was performed for 20 ns at 298 K for both nanocomposite systems. We prepared over 10 models in each case for statistically valid results.

### Deforming the PAN-SWNT nanocomposite

**Figure 3:**
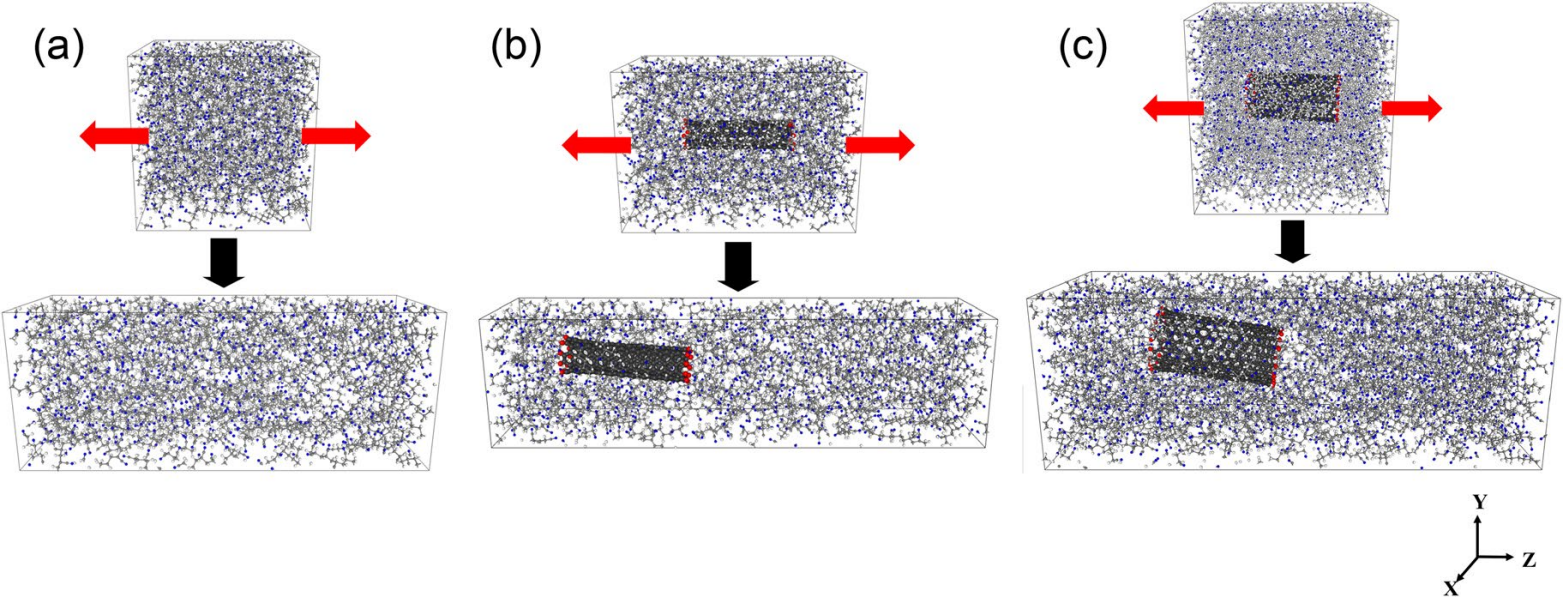
Extending (**a**) the PAN-matrix, (**b**) the PAN-SWNT_(5,5)_ nanocomposite, and (**c**) the PAN-SWNT_(10,10)_ nanocomposite. White, gray, and blue represent the hydrogens, carbons, and nitrogens of PAN, respectively; red and black represent the hydrogens and carbons of the SWNT, respectively.

In order to study the structural-evolution mechanism, the equilibrated PAN-matrix and the nanocomposite systems were uniaxially extended in the z-direction over 3.3 ns at 298.15 K at a stretching speed of 100 m/min, to the strain of 100%, under NPT conditions (Fig. 3). The critical aspect of this study is to show how and where the defective void structure is evolving in the early stage of the deformation, which can be shown in the relationship between the void formation and the corresponding mechanical properties. Following elongation, stress was calculated using Eq. (2)^44^:

2 $$P = \frac{{NK_{B} T}}{V} + \frac{{\mathop \sum \nolimits_{i}^{N\prime } r_{i} \cdot f_{i} }}{dV},$$

where N and N′ are numbers of particles, K_B_ is the Boltzmann constant, T is temperature, r_i_ and f_i_ are the positional and force vectors of atom i, respectively, and V is the volume. In particular, viral factors associated with interactions involving sums of pairs and their bond, angle, dihedral, improper, and k-space (long-range) energies, except kinetic energy, were derived from the second term in Eq. (2).

## Results and discussion

### Void analysis

**Figure 4:**
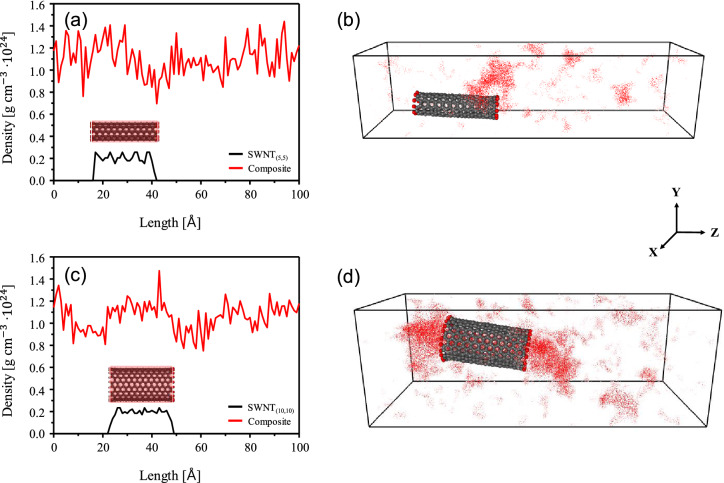
Density profiles of (**a**) the PAN-SWNT_(5,5)_ and (**c**) PAN-SWNT_(10,10)_ nanocomposites at the strain of 100%. Void profiles of the (**b**) PAN-SWNT_(5,5)_ and (**d**) PAN-SWNT_(10,10)_ nanocomposites at the strain of 100%. Red dots depict voids in each nanocomposite.

In order to clarify the void formation mechanism, polymer density profiles were investigated as shown in Fig. 4. Both nanocomposite systems exhibited a decreasing trend in density at the ends of SWNTs, and the extent of density decrease is more prominent in the nanocomposite containing large diameter SWNT_(10,10)_. In addition, the density of the polymer matrix at the SWNT body region remains similar to that of the polymer-alone region. This clearly suggests that void formation is predominantly taken place at the end of SWNT, and it may be further propagated upon stretching. The void formation and density profiles of the PAN-matrix is also shown in Fig. 2S with showing no significant localization of void formation and density. Jain et al.^19^ showed the elongated void structure at the end of PAN/vapor grown carbon nanofiber (VGCNF) nanocomposites where the VGCNFs diameter is on the order of hundred nanometers.

Figure 4b, d illustrates empty volume in nanocomposite systems, showing that voids are formed at the ends of the SWNTs, in particular, the significant void formation was observed in the PAN-SWNT_(10,10)_ nanocomposite as compared to the PAN-SWNT_(5,5)_ system. Voids were calculated by Connolly surface analysis^45^ in Materials Studio software^33^. The current simulation study on the microstructure evolution exhibits that the larger diameter CNTs possibly create the larger void, which may adversely affect the mechanical properties of nanocomposites, especially when the extensional flow is applied to the system. Based on the calculated mechanical properties, however, the difference of mechanical properties between the control PAN and PAN-SWNT nanocomposites is not significant because of the ductile nature of matrix polymer. In the case of nanocomposites based on the brittle matrix such as carbon, nonetheless, the fracture mechanics analysis by Griffith’s equation showed that the critical defect size attributed to carbon fiber failure is even on the order of nanometer scale^21^. Therefore, it is essential to understand process and microstructure development in order to achieve high-quality nanocomposites.

### Stress–strain analysis

**Figure 5:**
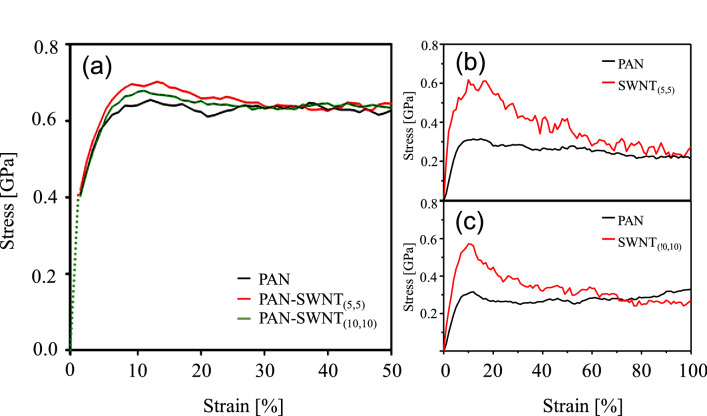
Stress-stain curves for (**a**) the PAN-matrix, and the PAN-SWNT_(5,5)_ and PAN-SWNT_(10,10)_ composite systems, (**b**) PAN-SWNT_(5,5)_ components and (**c**) PAN-SWNT_(10,10)_ components of the PAN-SWNT nanocomposite systems.

The stress–strain behavior of each system under extensional deformation is shown in Fig. 5a. All the stress–strain curves exhibit a linear elastic region with a strain level of up to about 10% followed by plastic deformation. It has been shown that polymer chains were generally well oriented in the vicinity of the SWNT in each nanocomposite; therefore, the mechanical properties of the nanocomposite, such as the Young’s and bulk moduli, were improved as the SWNT-containing nanocomposites were uniaxially elongated^6^. Figure 5a also exhibits that the nanocomposite systems endured higher stress at the same strain as compared to the control PAN. In addition, the stress of the nanocomposite containing SWNT_(5,5)_ was higher than other systems because SWNT with a smaller diameter can have higher elastic-energy densities compared to those of larger-diameter SWNTs.

Along with the evaluation of the composite properties, an effective stress transfer from matrix polymer to nanofillers is a critical parameter to be assessed for obtaining high-quality composite materials. Figure 5b, c show the calculated stress values applied to each component of the nanocomposites when they are subjected to elongational strain as high as 100%. It is interesting to note that both nanocomposites show effective stress transfer from matrix to SWNTs up until the yield point, which is about 10% of strain. Beyond the yield point, the stress transfer efficiency diminished continuously. In the case of PAN-SWNT_(5,5)_, the stress applied to SWNT_(5,5)_ was higher than that to the PAN matrix until 100% strain. On the other hand, PAN-SWNT_(10,10)_ system exhibited that the stress applied to SWNT_(10,10)_ was even lower than that to the PAN matrix beyond a strain level of 70%, suggesting that the stress transfer efficiency becomes very poor. In addition, the stress applied to the PAN matrix for the case of PAN-SWNT_(10,10)_ shows strain-hardening behavior, whereas that for PAN-SWNT_(5,5)_ remains almost constant throughout the entire strain level. This also indicates the relatively poor stress transfer efficiency for large diameter SWNT.

### Modulus analysis

The Young’s and bulk moduli^35,46^ were also calculated using a constant strain-minimization method, in which a small strain of 0.003% was simultaneously applied in all directions following energy minimization, as summarized in Table 2. The mechanical properties of all systems improved with decreasing SWNT diameter. If the SWNT is considered to be a solid cylinder, then its thickness correlates with the calculated elastic modulus when axial tension is applied to the composite. Consequently, the longitudinal Young’s modulus, E_11_, of the SWNT can be defined by Eq. (3)^35^:

3 $$E_{11} { } = { }\frac{F}{{2\pi Rt\varepsilon_{11} }}{ }$$

where F, R, t, and $\upvarepsilon _{{{11}}}$ represent force, radius, cylinder thickness, and axial strain, respectively. Because the wall thicknesses of the SWNT_(5,5)_ and SWNT_(10,10)_ are the same, Young’s modulus is only dependent on the radius of the SWNT. Therefore, the degrees of enhancement of the moduli of the nanocomposites are inversely proportional to their radii. In addition, according to Hooke’s law, stress σ is defined by σ = F/A, where A is the cross-section of the nanotube, and F is the force required to constrain the deformation. If the SWNT model is considered to be a uniform cylinder with negligible thickness, then stress is determined by the cross-sectional area of the SWNT^47^. Then, SWNTs with a smaller diameter is supposed to exhibit higher stress than those with a larger diameter. Therefore, as shown in Fig. 5a and Table 2, the stress in the linear elastic region, and the Young’s and bulk moduli of the SWNT_(5,5)_ nanocomposite were calculated to be higher as compared to those of the SWNT_(10,10)_-containing system. This is well agreed with the reported results as the SWNT with the smaller diameter can bear more stress against external deformation^41^.

**Table 2:** Young’s and Bulk moduli of the matrix and nanocomposite systems.

	Young’s modulus (GPa)	Bulk modulus (GPa)
PAN-matrix	3.51 ± 0.11	3.43 ± 0.39
PAN-SWNT_(5,5)_	4.76 ± 0.24	4.19 ± 0.25
PAN-SWNT_(10,10)_	4.41 ± 0.20	3.90 ± 0.17

### Structural analysis

**Figure 6:**
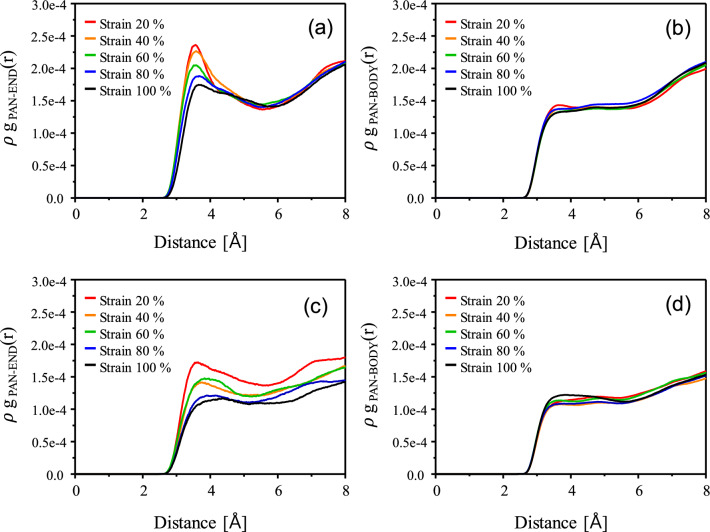
RDF curves for the SWNT_(5,5)_-PAN nanocomposite between (**a**) the ends of the SWNT_(5,5)_ and PAN, and (**b**) the body of the SWNT_(5,5)_ and PAN; and for the SWNT_(10,10)_-PAN nanocomposite between (**c**) the ends of the SWNT_(10,10)_ and PAN, and (**d**) the body of the SWNT_(10,10)_ and PAN, during extension.

Radial-distribution-function (RDF) analyses were performed for two pairs, namely between the nitrogen atoms of PAN and the carbon atoms at the SWNT ends, and the carbon atoms in the SWNT body and the nitrogen atoms of PAN in order to elucidate the structural evolution upon stretching. The RDF functions are given by Eq. (4):

4 $${\text{g}}_{A - B} {\text{(r) = }}{{\left( {\frac{{n_{B} }}{{4\pi r^{2} dr}}} \right)} \mathord{\left/ {\vphantom {{\left( {\frac{{n_{B} }}{{4\pi r^{2} dr}}} \right)} {\left( {\frac{{N_{B} }}{V}} \right)}}} \right. \kern-\nulldelimiterspace} {\left( {\frac{{N_{B} }}{V}} \right)}},$$

where $n_{B}$ is the number of particles located at a distance in a shell of thickness *dr* from particle A, $N_{B}$ is the number of B particles, and V is the volume, respectively. The ρg(r) was used for the direct comparison of the intensities of RDFs instead of g(r). The radial distribution functions (RDFs) for the uniaxially stretched PAN-SWNT nanocomposite models are presented in Fig. 6; in order to investigate structural changes, coordination numbers (CNs) were also calculated, as listed in Table 3. The RDF functions and CN values can provide mechanistic details of where defects are formed and how voids are propagated with increasing strain in the nanocomposite systems. Figure 6a, b show the RDF as a function of distance for PAN-SWNT_(5,5)_ at the end and body of the SWNT_(5,5)_, respectively. Figure 6c, d are the same for PAN-SWNT_(10,10)_. It can be noted from Fig. 6a, c that the intensity of the first peaks, which is between terminal carbon atoms and nitrogen atoms of surrounding PAN molecules, decreases upon stretching, suggesting that the population of surrounding PAN molecules decreases with increasing uniaxial stretching. Since PAN molecules move away from the terminal carbon atoms of the SWNT during elongation, this may be evidence of voids formation as reported in previous experimental literature which shows the formation of microcracks at terminal ends^48^. On the other hand, the first peaks that represent distribution intensity between the SWNT body and PAN as shown in Fig. 6b, d were essentially unchanged as the strain was increased. The CN values between the SWNT ends and the PAN in both systems decreased much more dramatically than those between the SWNT body and PAN as listed in Table 3. Based on the RDF results and the CN analysis, it can be concluded that structural changes in the PAN-SWNT_(5,5)_ and PAN-SWNT_(10,10)_ nanocomposite systems, upon elongation, are of void formation at the ends of the SWNT. It is commonly accepted that there is a strong van der Waals (vdW) interaction at the body region of SWNT due to the π-orbital misalignment, which is generated from the curvature of SWNT, inducing interfacial bonding with a polymer. Upon uniaxial stretching, polymer chains and CNT will be aligned along the stretching direction, and the difference in molecular mobility between polymer chains and CNT may undergo different stretching behavior. Therefore, the voids are formed at the end of CNT, not at the body of CNT.

**Table 3:** Coordination numbers (CNs) of the SWNT_(5,5)_-PAN and SWNT_(10,10)_-PAN nanocomposites.

		PAN-SWNT_(5,5)_		PAN-SWNT_(10,10)_	
		End	Body	End	Body
Strain (%)	20	2.02	1.28	2.08	1.36
	40	1.99	1.21	1.75	1.32
	60	1.84	1.19	1.77	1.34
	80	1.62	1.17	1.47	1.31
	100	1.44	1.11	1.35	1.40

## Conclusion

We investigated the correlation between deformation and structure development mechanisms of PAN-SWNT nanocomposites, and the corresponding mechanical properties using molecular dynamics (MD) simulation. Mechanical properties such as the Young’s and bulk moduli were improved by the SWNT addition. In addition, the SWNT with a smaller diameter provided good reinforcement and stress transfer efficiency. As the nanocomposites were deformed to the strain of 100%, the void formation was elucidated with the aid of RDFs and density profiles. Stress calculation applied to the SWNT and PAN matrix exhibited that the large diameter SWNT rapidly loses stress transfer ability at large strain, which mainly due to the formation of void formation. Although this study did not show the distinctive difference in mechanical properties between the control PAN and PAN-SWNT nanocomposite systems, it is expected that when PAN polymer is converted into a brittle carbon matrix, such elongated void structures will be detrimental to the mechanical properties. Therefore, tailoring microstructure will be the key parameter to obtain high-performance nanocomposite systems.

## Supplementary information

Supplementary figures.

## References

[1] Mo JH, Kim KC, Jang KS (2019). Well-dispersed carbon nanotube/polymer composite films and application to electromagnetic interference shielding. J. Ind. Eng. Chem..

[2] Bhattacharyya AR, Potschke P, Abdel-Goad M, Fischer D (2004). Effect of encapsulated SWNT on the mechanical properties of melt mixed PA12/SWNT composites. Chem. Phys. Lett..

[3] Li X (2015). Drawing dependent structures, mechanical properties and cyclization behaviors of polyacrylonitrile and polyacrylonitrile/carbon nanotube composite fibers prepared by plasticized spinning. Phys. Chem. Chem. Phys..

[4] Chae HG, Choi YH, Minus ML, Kumar S (2009). Carbon nanotube reinforced small diameter polyacrylonitrile based carbon fiber. Compos. Sci. Technol..

[5] Chae HG, Minus ML, Rasheed A, Kumar S (2007). Stabilization and carbonization of gel spun polyacrylonitrile/single wall carbon nanotube composite fibers. Polymer.

[6] Chae HG, Minus ML, Kumar S (2006). Oriented and exfoliated single wall carbon nanotubes in polyacrylonitrile. Polymer.

[7] Wang WJ, Murthy NS, Chae HG, Kumar S (2008). Structural changes during deformation in carbon nanotube-reinforced polyacrylonitrile fibers. Polymer.

[8] Nam TH (2015). Effects of CNT diameter on mechanical properties of aligned CNT sheets and composites. Compos. Part A Appl. Sci. Manuf..

[9] Thostenson ET, Chou TW (2003). On the elastic properties of carbon nanotube-based composites: modelling and characterization. J. Phys. D Appl. Phys..

[10] Frank E, Hermanutz F, Buchmeiser MR (2012). Carbon fibers: precursors, manufacturing, and properties. Macromol. Mater. Eng..

[11] Gill AS, Visotsky D, Mears L, Summers JD (2017). Cost estimation model for polyacrylonitrile-based carbon fiber manufacturing process. J. Manuf. Sci. Eng. Trans. ASME.

[12] Naito K, Yang JM, Tanaka Y, Kagawa Y (2012). The effect of gauge length on tensile strength and Weibull modulus of polyacrylonitrile (PAN)- and pitch-based carbon fibers. J. Mater. Sci..

[13] Meng JS, Zhang YY, Cranford SW, Minus ML (2014). Nanotube dispersion and polymer conformational confinement in a nanocomposite fiber: a joint computational experimental study. J. Phys. Chem. B.

[14] Gissinger JR, Pramanik C, Newcomb B, Kumar S, Heinz H (2018). Nanoscale structure-property relationships of polyacrylonitrile/CNT composites as a function of polymer crystallinity and CNT diameter. ACS Appl. Mater. Inter..

[15] Baughman RH, Zakhidov AA, de Heer WA (2002). Carbon nanotubes—the route toward applications. Science.

[16] Wang X (2011). Mechanical and electrical property improvement in CNT/Nylon composites through drawing and stretching. Compos. Sci. Technol..

[17] Saba N, Jawaid M (2018). A review on thermomechanical properties of polymers and fibers reinforced polymer composites. J. Ind. Eng. Chem..

[18] Jang D, Lee DS, Lee A, Joh HI, Lee S (2019). Opto-thermal technique for measuring thermal conductivity of polyacrylonitrile based carbon fibers. J. Ind. Eng. Chem..

[19] Jain R, Chae HG, Kumar S (2013). Polyacrylonitrile/carbon nanofiber nanocomposite fibers. Compos. Sci. Technol..

[20] Newcomb BA (2015). Processing, structure, and properties of gel spun PAN and PAN/CNT fibers and gel spun PAN based carbon fibers. Polym. Eng. Sci..

[21] Chae HG (2015). High strength and high modulus carbon fibers. Carbon.

[22] Mayo SL, Olafson BD, Goddard WA (1990). Dreiding: a generic force-field for molecular simulations. J. Phys. Chem..

[23] Jeon I-Y (2010). Sponge behaviors of functionalized few-walled carbon nanotubes. J. Phys. Chem. C.

[24] Lee SG (2012). Deswelling mechanisms of surface-grafted Poly(NIPAAm) brush: molecular dynamics simulation approach. J. Phys. Chem. C.

[25] Lee J (2017). A nanophase-separated, quasi-solid-state polymeric single-ion conductor: polysulfide exclusion for lithium-sulfur batteries. ACS Energy Lett..

[26] Doo G (2018). Tuning the ionomer distribution in the fuel cell catalyst layer with scaling the ionomer aggregate size in dispersion. ACS Appl. Mater. Inter..

[27] Lee JH (2018). Dispersion-solvent control of ionomer aggregation in a polymer electrolyte membrane fuel cell. Sci. Rep..

[28] Kwon SH, Lee SY, Kim H-J, Kim H-T, Lee SG (2018). Effect of binder content on Pt/C catalyst coverage in a high temperature polymer electrolyte membrane fuel cell. ACS Appl. Nano Mater..

[29] Mulliken RS (1955). Electronic population analysis on LCAO-MO molecular wave functions: 1. J. Chem. Phys..

[30] Perdew JP, Burke K, Wang Y (1996). Generalized gradient approximation for the exchange-correlation hole of a many-electron system. Phys. Rev. B.

[31] Plimpton S (1995). Fast parallel algorithms for short-range molecular-dynamics. J. Comput. Phys..

[32] Swope WC, Andersen HC, Berens PH, Wilson KR (1982). A Computer-simulation method for the calculation of equilibrium-constants for the formation of physical clusters of molecules—application to small water clusters. J. Chem. Phys..

[33] Materials Studio (Biovia, Dassault Systemes, San Diego, 2018).

[34] Lee SG, Brunello GF, Jang SS, Lee JH, Bucknall DG (2009). Effect of monomeric sequence on mechanical properties of P(VP-co-HEMA) hydrogels at low hydration. J. Phys. Chem. B.

[35] Lee SG, Brunello GF, Jang SS, Bucknall DG (2009). Molecular dynamics simulation study of P (VP-co-HEMA) hydrogels: effect of water content on equilibrium structures and mechanical properties. Biomaterials.

[36] Hurley RB, Tzentis LS (1963). Density of polyacrylonitrile. J. Polym. Sci. Part B Polym. Phys..

[37] Chiang R (1963). Crystallization and melting behavior of polyacrylonitrile. J. Polym. Sci. A.

[38] Shokrieh MM, Rafiee R (2010). On the tensile behavior of an embedded carbon nanotube in polymer matrix with non-bonded interphase region. Compos. Struct..

[39] Yang S, Yu S, Kyoung W, Han DS, Cho M (2012). Multiscale modeling of size-dependent elastic properties of carbon nanotube/polymer nanocomposites with interfacial imperfections. Polymer.

[40] Chae HG, Sreekumar TV, Uchida T, Kumar S (2005). A comparison of reinforcement efficiency of various types of carbon nanotubes in poly acrylonitrile fiber. Polymer.

[41] Niyogi S (2002). Chemistry of single-walled carbon nanotubes. Acc. Chem. Res..

[42] Natsuki T, Tantrakarn K, Endo M (2004). Effects of carbon nanotube structures on mechanical properties. Appl. Phys. A.

[43] Mori H, Hirai Y, Ogata S, Akita S, Nakayama Y (2005). Chirality dependence of mechanical properties of single-walled carbon nanotubes under axial tensile strain. Jpn. J. Appl. Phys..

[44] Thompson AP, Plimpton SJ, Mattson W (2009). General formulation of pressure and stress tensor for arbitrary many-body interaction potentials under periodic boundary conditions. J. Chem. Phys..

[45] Connolly ML (1983). Analytical molecular-surface calculation. J. Appl. Crystallogr..

[46] Theodorou DN, Suter UW (1986). Atomistic modeling of mechanical-properties of polymeric glasses. Macromolecules.

[47] Cornwell CF, Wille LT (1997). Elastic properties of single-walled carbon nanotubes in compression. Solid State Commun..

[48] Coleman JN, Khan U, Gun'ko YK (2006). Mechanical reinforcement of polymers using carbon nanotubes. Adv. Mater..

