# Supplementary material for: Defect structure evolution of polyacrylonitrile and single wall carbon nanotube nanocomposites: a molecular dynamics simulation approach
Source: Sci Rep. 2020 Jul 16;10:11816. doi: 10.1038/s41598-020-68812-7 (PMC7366919; doi:10.1038/s41598-020-68812-7)
Supplement: Supplementary file 1 — Supplementary figures. [file 41598_2020_68812_MOESM1_ESM.docx]

**Supporting Information**

**Defect structure evolution of polyacrylonitrile and single wall carbon nanotube nanocomposites:**

**A molecular dynamics simulation approach**

So Jeong Heo^1,2^, Kwang Ho Kim^3^, Byungchan Han^4^,

Han Gi Chae^1,*^ and Seung Geol Lee^2,*^

*^1^* *School of Materials Science and Engineering, Ulsan National Institute of Science and Technology, 50 Unist-gil, Ulsan, 44919, Republic of Korea*

*^2^ Department of Organic Material Science and Engineering, Pusan National University, 2, Busandaehak-ro 63beon-gil, Geumjeong-gu, Busan, 46241, Republic of Korea*

^3^ *School of Materials Science and Engineering, Pusan National University, 2, Busandaehak-ro 63 Beon-gil, Geumjeong-gu, Busan, 46241, Republic of Korea*

*^4^ Department of Chemical & Biomolecular Engineering, Yonsei University, 50 Yonsei-ro, Seodaemun-gu, Seoul 03722, Republic of Korea*

*Corresponding authors:

E-mail: [seunggeol.lee@pusan.ac.kr](mailto:seunggeol.lee@pusan.ac.kr) (Seung Geol Lee)

E-mail: [hgchae@unist.ac.kr](mailto:hgchae@unist.ac.kr) (Han Gi Chae )

**Figure 1S.** The parital charges of (a) PAN and (b) SWNT models. White, gray and blue colored circles represent hydrogen, carbon and nitrogen atoms, respectively.

**Figure 2S.** (a) Density profiles of the PAN-matrix at strain of 100%. (b) Void profiles of the PAN-matrix at strain of 100%. Red dots depict voids.
